# Guessing PINs, One Partial PIN at a Time

**DOI:** 10.3390/e24091224

**Published:** 2022-09-01

**Authors:** Ashley Sheil, David Malone

**Affiliations:** Department of Mathematics and Statistics and the Hamilton Institute, Maynooth University, R51 A021 Co. Kildare, Ireland

**Keywords:** guessing, PINs, partial passwords, partial PINs

## Abstract

Entering digits of a personal identification number (PIN) is a common form of authentication. One variant of this scheme is to request the digits from a random subset of positions, which is sometimes called a *partial PIN*. In this paper we consider strategies for guessing the PIN when a partial PIN scheme is in use, which allows the quantification of the strength of this mechanism. We suggest several strategies for guessing the PIN under the assumption that the organisation assigns PINs randomly and requests random positions from the PIN at each login. We present analytic and simulation results from the different strategies and explore their performance when guessing different sizes of PIN and requested subset. We find that the most effective strategies have a reasonable chance of recovering a PIN in tens to hundreds of guesses.

## 1. Introduction

PINs are commonly used for bank cards and unlocking your phone. For a four-digit number there are 10,000 different possible combinations to check. One variation of the simple PIN is to request a subset, or *partial password*, of digits from a longer PIN. This has the advantage that an evesdropper who sees a single login does not learn the full PIN [1]. This scheme is commonly used by banks in some countries (see Section 2.1 for examples). However, these partial PINs can still be guessed. To do this manually, you would record numbers that were asked and any correct digits found. By beginning with a list of all possible PINs and deleting those that do not match as you progressed, the gradually reducing list would increase the probability of correctly guessing.

We are motivated to study guessing such PINs for two reasons. First, there are situations where in-the-wild attacks using such guessing are practical. An example scenario sees an attacker, Craig, who has access to Alice’s phone and attempts to gain access to her online banking account, which is protected by a partial-PIN scheme. Craig plays a long game, attempting to guess Alice’s banking PIN by guessing the partial PIN; say he has a few attempts each day in which he tries to guess the correct partial PIN but stops before being locked out. The next day he retries, each time ruling out incorrect guesses and crossing them off the list of possible combinations. If he gains access with a correct partial PIN guess he now has those digits of the full PIN and can eliminate all combinations which do not have those digits in the correct positions. Eventually, with enough patience, Craig will guess the whole PIN.

As described above, Craig has access to Alice’s device, which might be possible if Craig is, for example, a co-worker or is caring for Alice. Hence, any blocking mechanism based on device, location or IP address is unlikely to be effective. However, we can also consider weaker versions of the attack, where Craig does not have regular access to the device but might share accommodation with Alice, and so still share a location and IP address. A weaker attack still might see Craig attacking a pool of users after an online leak of information, and with access to a large pool of IP addresses to make guesses from. In each of these cases, the number of guesses required is an important factor in the success of the attack, while implementing a blocklist or rate limit based simply on IP address would incur significant practical issues, and it would be challenging to differentiate low-rate guessing from accidentally misentered digits.

Second, we believe the theoretical strength of these partial-PIN mechanisms has not been quantified, and we aim to do this in terms of number of guesses required to determine the secret (i.e., the full PIN). This is analogous to characterising the number of guesses required to identify user-chosen passwords or machine chosen secrets [2]. We believe it is important to offer some insight into the security of these partial PINs, as they remain in use as a factor in banking authentication. We choose the number of guesses to recover the full PIN as the metric of interest, as the use of partial PINs is motivated by protecting the full PIN from an attacker. However, similar techniques can be applied to study the number guesses before the first successful login.

In this paper, we will explore four strategies for guessing a PIN and study them via analysis and simulation to see how the different strategies perform and understand the effective strength of the partial-PIN mechanism. Section 2 reviews previous work in this area. Section 3 describes our strategies, with Section 4 giving some mathematical analysis of the strategies. Section 5 shows guessing performance of the strategies and Section 6 discusses these results in context. Finally, Section 7 concludes and discusses further work in the area.

## 2. Previous Work

### 2.1. PINs as a (Second) Factor in Banking

PINs in banking made their debut in the Barclays-De La Rue system in 1967, initially with 6 digits and subsequently reducing to 4 when the wife of the leader of the engineering team, John Shepard Barron, was said to have been unable to recall six random digits [3]. Banks began allowing customer-chosen PINs in the 1980s as a marketing initiative [3]. Some banks took on the user-chosen PIN idea, but have since discovered security flaws in this. Markert et al. demonstrate the flaw in their paper [4]: humans tend to use patterns of numbers to make their PINs easier to recall.

A common practice in some jurisdictions is that, when banking online, the bank will request a random subset of a customer’s PIN. Different banks may request different sized subsets and may use different sized PINs. With the development of strong authentication, partial PINs are being combined with other forms of authentication. The second Payment Services Directive, or PSD2, is a European law that came into effect in September 2019. This law aims to make online banking more secure by adding strong customer authentication (SCA) [5,6]. This may change the use of partial PINs for a number of reasons, including the availability of multi-factor authentication methods (MFA) and challenges in storing hashed partial passwords [7]. Consequently, banks are changing their login procedures, which depending on the individual bank may vary for e.g., Allied Irish Bank (AIB) will now require their customers to log in online with their full personal access code (PAC), where before you were required to input a subset of your PAC. AIB appear to be the only Irish bank that have abandoned partial PIN outright however, other banks like Bank of Ireland (BOI) have kept their partial PIN but added other forms of authentication. The Credit Union requires a partial PIN for its banking app as well as Trustee Savings Bank (TSB). In the UK the Hongkong and Shanghai Banking Corporation (HSBC) use a partial password and a question, Santander uses partial PIN and a partial password. When banking online via a laptop, your smart phone provides second factor authentication and would appear to be more secure than using your smart phone alone to bank online. For online purchasing you are required to authenticate with a second factor SMS or your banking app. Others require bio-authentication like facial recognition or a third verification like a personal question.

There is no particular reason why partial PINs or passwords should be restricted to banking. Indeed, Symantec’s Advanced Authentication product, which provides authentication support for mobile and web applications, includes support for partial passwords (See, for example, https://techdocs.broadcom.com/us/en/symantec-security-software/identity-security/advanced-authentication/9-1/release-notes-9-1.html (accessed on 28 August 2022). However, in practice, common examples of its use seem to be in the banking sector.

### 2.2. Guessing of PINs and Passwords

Password guessing is a popular research topic in the area of banking and online security, however most research is aimed at user-chosen PINs. Bonneau et al. investigate the security implications of human selection and management of PINs [8], based on the leaked data set ‘RockYou’, from which they discovered that 1234 was the most common user-chosen four-digit PIN. Markert et al. also availed of this data set when investigating user-chosen PINs [4]. Birth dates were discovered to also be a popular choice for PINs (especially for four and six-digit PINs), as were repeated digits. Bonneau et al. advise users not to use PINs based on date of birth. They also advise those banks that do not employ blocklists of weak PINs to immediately do so [8]. Bentley and Mallows postulate that “Humans tend to choose secrets in nonrandom and repeated patterns” [9].

In light of this research, randomly assigned PINs appear a more logical approach for security, and some banks appear to follow this approach. Research in the area of guessing sets of random numbers, such as PINs, is harder to find. The closest previous work done in this area is by Kuhn where he uses probability to guess randomly generated PINs for ATM cards [10]. Knuth focuses on the game Mastermind, where rather then numbers, you are guessing colours in a sequence of four coloured pegs out of six possible colours, hence there are $6^{4}=1296$ possible combinations [11]. Focardi and Luccio follow on Knuth’s idea of solving the Generalized Mastermind Game problem and link it with guessing bank PINs [12]. They set out a framework for guessing games which we will use to describe our problem. In terms of exhaustive guessing methods, which we explore in our paper, Chiasson and Oorschot explore methods of guessing passwords relating to password expiration policies [13].

Aspinal and Just also investigate partial passwords, both character and numerical [1]. They concede that this is an area that has received less attention than others. They, again, are looking at user-chosen passwords and PINs. They also avail of the RockYou data set in their study, where they find that with “6 guesses, an attacker can respond correctly to 2-place challenges on 6-digit PINs with a success rate of 30%” and “Recording up to 4 runs, an attacker can succeed over 60% of the time”. They also record how quickly they can guess the full password after learning a subset of the password.

In our paper, we look at how quickly a PIN can be guessed, which is similar to Aspinal and Just, with key differences being that we consider randomly assigned PINs and the possibility of tracking all information learned by guessing. The latter is important in the effective guessing of uniformly assigned random PINs, as no information about non-random human choices can be exploited. In their terms, it corresponds to designing an *adaptive projection dictionary* attack, suggested in their future work.

## 3. Strategies for Guessing a PIN

Assume that the bank assigns an *n*-digit PIN to a user uniformly at random, for use for future logins. For simplicity of exposition, we use n=4 for our examples in this section, so there are 10,000 possible PINs.

When a user (or attacker) attempts to log in, the bank picks *m* positions from the *n* digits of the PIN. In these examples, we work with m=2. The user must provide these digits. If they provide them correctly, they are given access to the system. If they do not provide them correctly, they are refused access. We assume that repeated login attempts result in a new random selection of positions from the PIN. Further login attempts can then be made.

We denote these problems as ${}^{n}C_{m}$. In real systems, login attempts are often throttled or rate-limited, but here we assume that many guesses can be made in order to assess how many guesses are required before an attack can be successful. This might be achieved by an attacker rate-limiting their guesses or interleaving them with legitimate logins by a valid user to avoid lockout. If attempts are limited, then our results can be used to understand the chance of success after a particular number of guesses.

### Attacker Strategies

We suppose that the attacker begins with a list of all possible PINs, which we call the PIN list. The attacker can use this list to inform their choice of guess, and we call this a strategy. After each guess, the attacker updates the PIN list. If the attacker guesses the m=2 chosen positions correctly, all four-digit numbers that do *not* have this combination of digits in the requested positions are deleted from the PIN list. If the guess is wrong, then all PINs *with* this combination of digits are deleted from the PIN list. In this way, if the PIN list reduces in length at each guess, the attacker will eventually find the correct PIN.

In abstract terms, following the notation for a general guessing game in [12], guessing the PIN can be described as follows:

1. The bank chooses the secret *s*, which in our case is the PIN.
2. The PIN list begins as the set of possibilities, *S*, and ranges from 0000 to 9999.
3. The *i*^th^ guess $g_{i}$, for a random subset of m=2 digits of the PIN.
4. The response to the guess *i*^th^ guess $=r_{s,i}\left(g_{i}\right)$ depends on the secret *s* and the guess $g_{i}$ and the random subset selected. In our case, this is a successful login if the guessed digits match and a login failure otherwise.
5. The list of remaining PIN numbers is $S_{i}=\left\{v\in S:r_{v,i}\left(g_{i}\right)=r_{s,i}\left(g_{i}\right)\right\}$.
6. The secret *s* must be in the intersection of the sets $S_{i}$.

Note, an attacker can incorporate additional information, such as an eavesdropped login attempt, by starting with a different initial PIN list *S*.

In this paper we consider four strategies for choosing the guesses. Pseudocode for each is shown in in Algorithm 1).

**Max**  At each guess, this method uses the PIN list to find the frequency of combinations of digits in the positions requested by the bank. The combination with the highest frequency is guessed, with ties broken randomly.
**Educated Guess**  This method looks at the distribution of digit combinations for the positions requested by the bank, and chooses a combination according to this distribution. For example, if the second and third digit are requested and 50% of PINs remaining have 77 in this position, while 25% have 78 and 25% have 79, then 77 will be chosen 50% of the time, etc. In practice, this can be achieved by choosing one PIN at random from the remaining PIN list and using the requested digits.
**Round Robin**  The Round Robin method starts by trying 0 for each position. On the next guess requesting this position, it will guess 1, then 2 and so on, wrapping at 9. This method does not use the PIN list to choose its digits. Note, in the ${}^{n}C_{n}$ case it will usually not succeed; for example, if guessing 4 digits from 4, it will only guess 0000, 1111, …, 9999. For ${}^{n}C_{m}$ problems where m<n and random (strict) subsets of digits are being selected, it will eventually guess every combination.
**Random**  The random method chooses random numbers for each of its guesses. It will eventually guess all combinations with probability 1. As with Round Robin, it does not use the PIN list to guess and may repeat guesses.

Each strategy has been coded (see pseudocode in Algorithm 1), to allow simulation of its performance. We expect that the last two strategies will not usually be competitive, however we include them for two reasons. First, they offer a useful comparison. Second, since they do not use the PIN list to make choices, they avoid many operations on the PIN list which is initially of size $10^{n}$. For large *n*, maintaining this list might be prohibitive.

**Algorithm 1** Pseudocode for Strategies for ${}^{n}C_{m}$ problems.
1:PIN[i]← i^th^ remaining PIN2:bank[i]← i^th^ position requested3: 4:
**procedure** Max
5:      $\mathrm{count}\leftarrow \mathrm{zero}(10^{m})$6:      **for each** p in PIN[] **do**7:          v← digits of p at bank8:          count[v]++9:      **end for**10:  **return** ${arg max}_{v}\mathrm{count}\left[v\right]$11:
**end procedure**
12: 13:
**procedure** EducatedGuess
14:      p← random PIN from PIN[]15:      v← digits of p at bank16:      **return** v17:
**end procedure**
18: 

19:
**procedure** Random
20:      p← random from 0 – $10^{n}-1$21:      v← digits of p at bank22:      **return** v23:
**end procedure**
24: 25:
**Init:**
      RRdigit[]←zero(n)26:
**procedure** RoundRobin
27:      v←028:      **for each** d in bank[] **do**29:          v←10×v+RRdigit[d]30:          RRdigit[d]++mod1031:      **end for**32:      **return** v33:
**end procedure**
34: 35: 


## 4. Analysis

In this section, we briefly consider some analysis that can be carried out of the PIN guessing strategies.

### 4.1. Stepwise Optimality of Max Strategy

It is possible to analyse the guessing process at each step. Suppose we are at a point in the guessing process where *N* entries remain on the PIN list. The bank asks about *m* positions in the PIN. We let $N_{d_{1}\ldots d_{m}}$ be the number of entries in the PIN list with the digit $d_{1}$ in the first requested position, digit $d_{2}$ in the second requested position, and so on. If PINs are assigned uniformly at random, then the chance of guessing the digits $d_{1}\ldots d_{m}$ correctly will be $p_{d_{1}\ldots d_{m}}=N_{d_{1}\ldots d_{m}}/N$.

Using this probability, we can aim to choose the digits that maximise the chance of particular outcomes. For example, to aim to maximise the chance of a successful login on the next guess, we choose the digits $d_{1}\ldots d_{m}$ with the highest frequency, corresponding exactly to our Max strategy.

One might also aim to minimise the expected number of entries that remain on the PIN list, thus reducing its size as quickly as possible. If we choose $d_{1}\ldots d_{m}$ and we are correct, then just $N_{d_{1}\ldots d_{m}}$ will remain after this guess. If we are incorrect, then all combinations not matching these digits will remain, i.e., $N-N_{d_{1}\ldots d_{m}}$ combinations. Thus, to achieve this aim we choose the digits to minimise

$$p_{d_{1}\ldots d_{m}}N_{d_{1}\ldots d_{m}}+\left(1-p_{d_{1}\ldots d_{m}}\right)\left(N-N_{d_{1}\ldots d_{m}}\right)=N\left(p_{d_{1}\ldots d_{m}}^{2}+{\left(1-p_{d_{1}\ldots d_{m}}\right)}^{2}\right).$$

Note that this function is quadratic in $p_{d_{1}\ldots d_{m}}$ with a minimum at 0.5. Thus, to minimise the size of the remaining PIN list, we should choose the combination of digits that has frequency closest to half the number of remaining PINs. As it is unusual to have one combination of digits to be the majority of the PIN list, this aim will also usually correspond to the Max strategy.

A slightly more cautious aim might be to select the digits that minimise the remaining size of PIN list in the worst case, regardless of whether it is a correct guess or not. Here, we choose the digits that minimise $\max(N_{d_{1}\ldots d_{m}},N-N_{d_{1}\ldots d_{m}})$. This is a piecewise linear function in $N_{d_{1}\ldots d_{m}}$, with a minimum at N/2. So again, we should choose the combination of digits that is closest to half the remaining digits. As noted above, this aim usually corresponds to the Max strategy.

Interestingly, the three above aims usually result in the same action as our Max strategy, providing no single combination of digits is in the majority. Consequently, we expect that the Max strategy should perform well in terms of both reducing the size of the PIN list and achieving successful logins. Note that each of these aims are greedy, in the sense that they optimise gains one step ahead.

### 4.2. Equivalence of Strategies for ${}^{n}C_{n}$ and ${}^{n}C_{1}$ problems

Note, that in the ${}^{n}C_{n}$ case all PINs start as equally likely. After each guess, we either guess correctly (and stop as n=m) or we guess incorrectly and eliminate one possible PIN from the list. Thus, the Max and Educated Guess strategies are presented with a list of equally likely options, and so choose effectively randomly from the list. This means that in the ${}^{n}C_{n}$ case, Max and Educated Guess perform equally well.

Similarly, in the ${}^{n}C_{1}$ case, we effectively have independent PIN lists for each position, as a guess about one position tells us nothing about the other positions. Each guess for each position is either correct or removes a digit, and either way we are left with a list of equally likely possibilities for this position. So, when Max or Educated Guess come to make choices, they will again behave in the same way. In fact, the Round Robin strategy will also perform in the same way, as even though it does not use the PIN list, in the ${}^{n}C_{1}$ case, it will not repeat any guesses for a position, effectively choosing at random from the remaining digits because the PIN was assigned randomly.

### 4.3. Performance of Max and Random Strategies for ${}^{n}C_{n}$ and ${}^{n}C_{1}$ problems

As we noted, in the case where m=n, analysis of the guessing problem is greatly simplified. In this case, each guess either results in the identification of the PIN or the elimination of a single PIN from the PIN list. As we show in the Appendix A, this makes it possible to calculate the distribution of the number of guesses explicitly for both the Max and Random strategies, using standard probabilistic techniques in Equations (A1) and (A6). Via our observation in Section 4.2, we note the analysis also covers the Educated Guess strategy.

Similarly, in the case where m=1, we are essentially faced with guessing a sequence of independent digits, where guessing one digit does not influence what we know about the others. Note that after *k* guesses, the number of times we will have guessed each digit will follow a multinomial distribution. By using this observation, and the results for the ${}^{1}C_{1}$ problem, we obtain explicit expressions for the distribution of guesses when using Max and Random strategies for the ${}^{n}C_{1}$ problems, see Equations (A3) and (A8) in the Appendix A. Via our observation in Section 4.2, we see that the analysis will also cover the Educated Guess and Round Robin strategies. We note that these expressions can become unwieldy when applied for larger numbers of guesses.

Using these distributions, we can also calculate the expected number of guesses required. This requires an infinite sum, but we show how to bound the infinite sum using a finite number of terms (see Equation (A11)).

**Figure 1:**
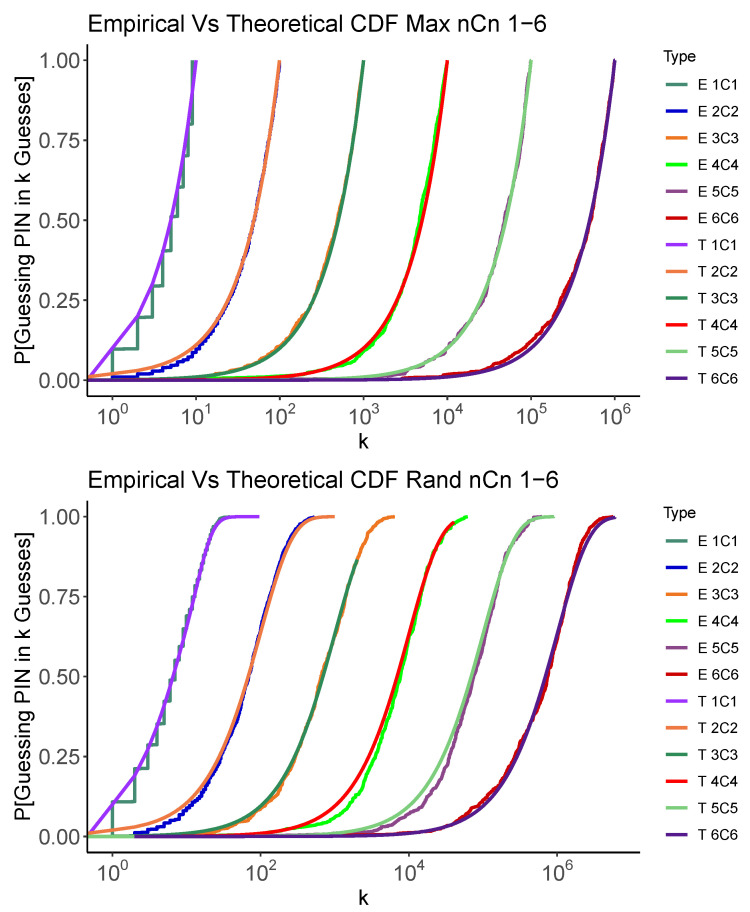
Simulated and analytic performance of the Max (**above**) and Random (**below**) strategies for ${}^{n}C_{n}$. Each graph shows the CDF for the number of guesses, *k*, required to recover the PIN.

**Figure 2:**
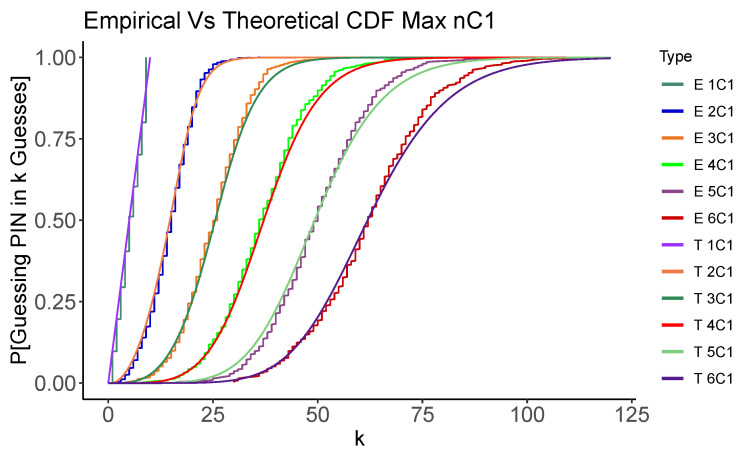

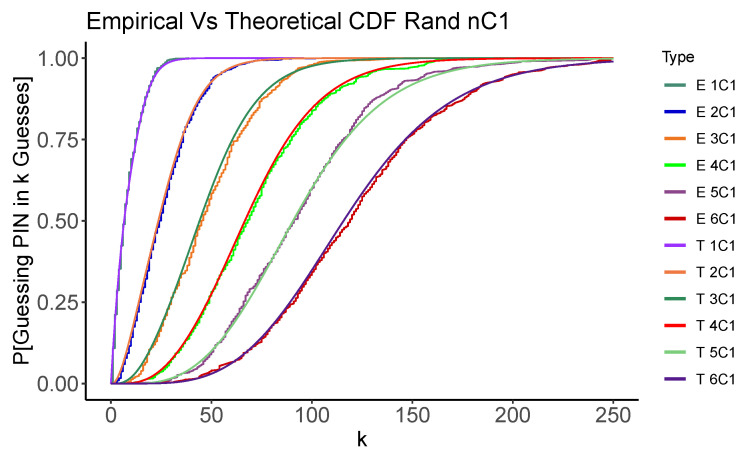
Simulated and analytic performance of the Max (**top**) and Random (**bottom**) strategies for ${}^{n}C_{1}$. Each graph shows the CDF for the number of guesses, *k*, required to recover the PIN.

Figure 1 and Figure 2 graphically show how the empirical simulations results generated by the code mentioned in Section 3 closely match the theoretical analysis results conducted in the Appendix A for ${}^{n}C_{n}$ and ${}^{n}C_{1}$ problems using the Random and Max strategies. The code carries out 500 random trials to guess a PIN selected at random. In each case, n=1…6, we show the Cumulative Distribution Function (CDF) for the number of guesses required to recover the PIN. While we will show the details of these results in the next section, we can see that the two methods of estimating the distribution of number of guesses concur. We also see that, as expected, the Max strategy also tends to use fewer guesses than the Random strategy. This gives us confidence that, at least in these simplified cases, our analysis and code are operating as expected.

## 5. Results

**Figure 3:**
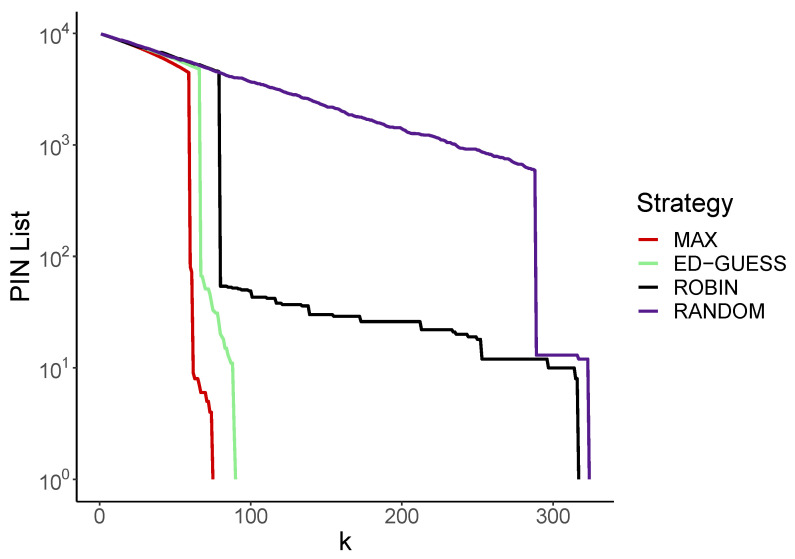
Length of the PIN list after *k* guesses for a single run for each strategy (guessing m=2 from n=4, log scale).

As an initial example, consider a single run of the ${}^{4}C_{2}$ problem using each strategy. In Figure 3 the y-axis displays the remaining number of entries on the PIN list (log scale), and the number of guesses taken is shown on the x-axis.

We see that for each strategy, the number of entries on the PIN list decreases, with occasional sudden drops. These drops correspond to a successful login attempt, which typically removes many entries from the PIN list. Looking at the Max strategy, this happens quite quickly at around 70 guesses, followed by the Educated Guess method, and then the Round Robin method. Lastly the Random method has a success after about 300 guesses. When the lines reach y=1, the full PIN has been discovered, which occurs in the same order as the drops: Max, Educated Guess, Round Robin and finally Random.

As we will see, this ordering is typical of the general case, as is the larger gap between the Max/Educated guess and the Random/Round Robin strategies that make their guesses independently of the PIN list.

### 5.1. Results for Various Partial PIN

In the previous example, we considered a single ${}^{4}C_{2}$ problem, where we provided two digits from four. In this section, we look at the performance of the strategies on other PIN sizes over many trials.

**Figure 4:**
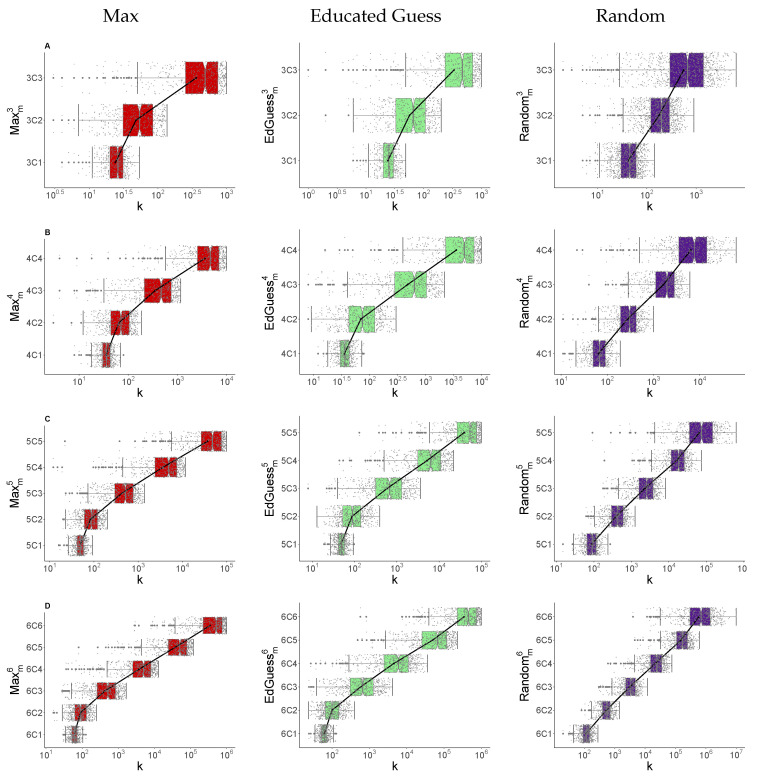
Summary of number of guesses, *k*, requied for Max/Educated Guess/Random strategies, Row A: n=3 for m=1,2,3, Row B: n=4 for m=1,2,3,4, Row C: n=5 for m=1,2,3,4,5 and Row D: n=6 for m=1,2,3,4,5,6, 500 runs. (Box 1st/3rd quartile, whisker ± 1.5 IQR).

**Figure A2:**
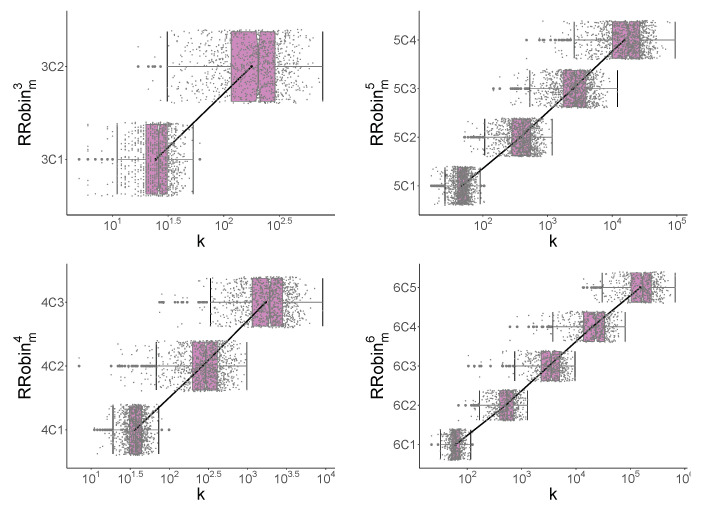
Summary of number of guesses, *k*, for Round Robbin strategies, n=3 for m=1,2, n=4 for m=1,2,3, n=5 for m=1,2,3,4 and n=6 for m=1,2,3,4,5, 500 runs. (Box 1st/3rd quartile, whisker ± 1.5 IQR).

Figure 4 summarises the results for 500 runs of each ${}^{n}C_{m}$ problem, using a box plot to show the number of guesses for different strategies and different values of *n* and *m*. The top row (row A) shows results for n=3 and the bottom row (row D) shows results for n=6. Within the plot for each *n* value, results for subsets of size m≤n are shown. We show results for each of the Max, Educated Guess and Random strategies. Here, for ease of reading, we omit the results for the Round Robin strategy until the Appendix A (see Figure A2), as its performance is broadly similar to other cases. Round Robin results are similar to Random except in the ${}^{n}C_{1}$, where it follows Max/Educated Guess (as discussed in Section 4.2) and the ${}^{n}C_{n}$ case, where it usually fails (as discussed in Section 3). Each dot represents the number of guesses required to find the full PIN for a single run, with boxes showing the first and third quartile. Notches give a 95% confidence interval for the median. A line joining means has also been included to highlight how the mean changes as *m* increases.

For example, consider the ${}^{3}C_{m}$ guessing problem in the top row (row A). It becomes harder to guess the PIN as *m* increases for all strategies. We can also see that the performance of the Max and Educated Guess strategies is broadly similar, with Max having a slight edge. The Random strategy lags, with the smallest relative gap for ${}^{3}C_{3}$.

Looking at the left edge of the box gives the number of guesses required for a 25% success rate in determining the full PIN. With the Max strategy, we obtain the PIN with a 25% success rate with approximately 20, 30 and 250 guesses for ${}^{3}C_{1}$, ${}^{3}C_{2}$ and ${}^{3}C_{3}$ respectively.

Similar results are presented for ${}^{n}C_{m}$ for n=4,5,6 in rows B, C and D, respectively. We see that the ordering of the schemes and increase with *m* is broadly maintained. Interestingly, for the Max strategy, if we keep *m* fixed and increase *n*, we see a relatively small increase in the median number of guesses. We also see that the number of guesses required for a 25% chance to obtain the full PIN can be surprisingly small. For example, in the ${}^{4}C_{2}$ case it is a little under 50 guesses and the ${}^{6}C_{3}$ it is a little under 300 guesses.

### 5.2. Comparison of Strategies

**Figure 5:**
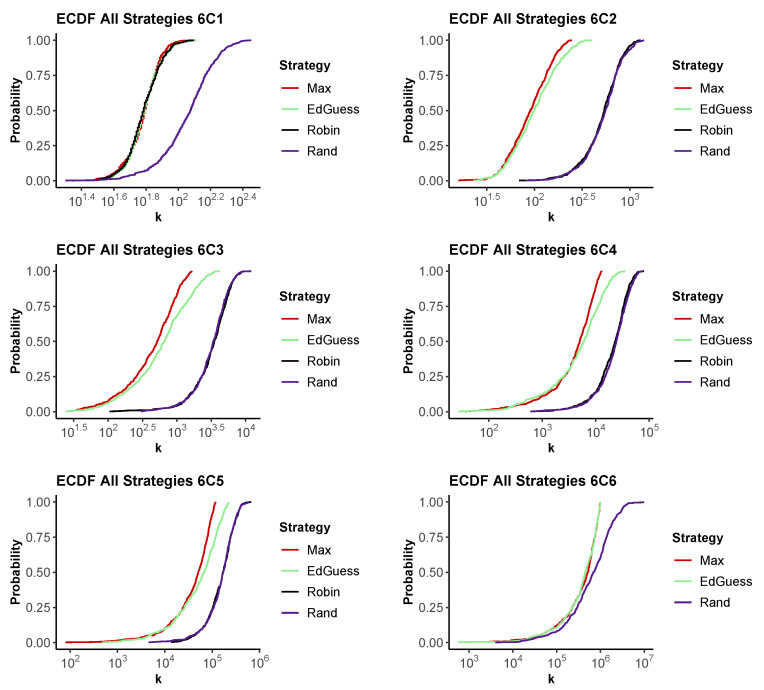
The empirical CDF for the number of guesses, *k*, required to recover a PIN for a ${}^{6}C_{m}$ problem for each strategy. Top row m=1,2,3. Bottom row m=4,5,6. 500 runs.

To allow a comparison of the performance of different strategies, Figure 5 shows the results for ${}^{6}C_{m}$ problems, plotting the CDF for the strategies on a single graph. The further left the CDF for a strategy, the more quickly it is likely to recover the full PIN. These graphs show several interesting features that are observed in our results for ${}^{n}C_{m}$ for other values of *n*.

First, observe that as expected in the ${}^{6}C_{1}$ case, we see that Max, Educated Guess and Round Robin all have similar performance, with a 50% chance of recovering the PIN in around 60 guesses. Random lags considerably once more than a handful of guesses are made.

Again, as expected from Section 4.2, we see that Max and Educated Guess perform equally well for ${}^{6}C_{6}$ problems, always guessing the pin in less than 1,000,000 guesses. The random scheme lags slightly initially, with a long tail where it is unlucky and repeatedly makes incorrect guesses.

Between the extremes of ${}^{6}C_{1}$ and ${}^{6}C_{6}$, we see that Round Robin and Random perform similarly, both lagging Max and Educated Guess considerably. For smaller numbers of guesses, Educated Guess and Max behave similarly. However, Max makes more efficient use of what it has learned if the number of guesses is large.

### 5.3. Varying n and m in ${}^{n}C_{m}$

In the previous subsection, we compared the performance of our strategies. However, it is also reasonable to ask how the difficulty in the guessing problem changes as we vary *n* and *m* in more detail. If we fix *m* and increase *n*, the impact seems clear: increasing *n* increases the number of initially unknown digits without changing the difficulty of the individual guesses. Indeed, this matches what we see in practice.

If we fix *n* and increase *m*, the situation is more complex. Increasing *m* increases how much we learn on each step, while also making the probability of a successful guess less likely. As a successful guess usually provides the most information, it is not immediately obvious how these factors trade off against one another for different measures of difficulty.

**Figure 6:**
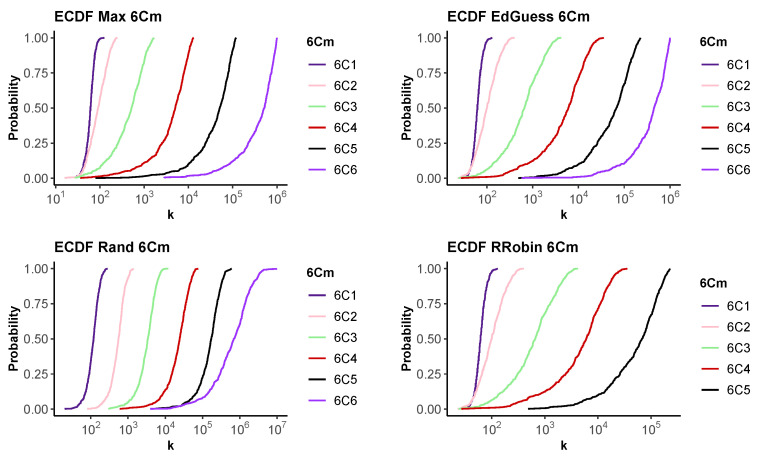
The empirical CDF for the number of guesses, *k*, required to recover a PIN for a ${}^{6}C_{m}$. Top row Max, Educated guess. Bottom row Random and Round Robin. 500 runs.

Figure 6 shows the results of fixing n=6 while varying *m* for each of our four strategies. We present the ECDF for various values of *m* on a single graph for each strategy. We see that as *m* increases, the graphs move to the right. This indicates that an increase in the number of guesses required to achieve any particular success rate, indicating stochastic dominance. We conclude that increasing *m* increases the difficulty of recovering the PIN by guessing.

## 6. Discussion

In designing our strategies, our analysis indicated that the Max strategy should be effective in discovering the PIN most quickly, and this has been borne out. However, the performance of the Educated Guess strategy can approach the Max strategy. By consulting Algorithm 1, we can see that while Educated Guess also uses a PIN list, its implementation is simpler and so less computationally complex than the Max strategy. The Round Robin and Random strategies do not require a PIN list to generate guesses, however their performance is significantly worse in most cases.

If *n* is fixed and *m* is increased, the difficulty of guessing increases. Going back to Figure 4, we observe approximately convex behaviour for the mean/median of the strategies on a log scale. In fact, if we restrict our attention to the ${}^{n}C_{m}$ problems with m>1, the observed pattern is almost (log-)linear. We conjecture the existence of this convex behaviour in general. If confirmed, this behaviour could be used to bound the guessing cost of ${}^{n}C_{m}$ in terms of ${}^{n}C_{n}$ and ${}^{n}C_{1}$, cases we have provided an analysis of in the Appendix A. Alternatively, techniques such as Large Deviations might be used to give asymptotic estimates, as they have been for the guesswork of various distributions [14,15,16].

The Max strategy seems to require relatively little extra effort to guess a ${}^{n}C_{m}$ as *n* increases and *m* is fixed. This possibly indicates efficient use of cross-position information learned as guesses are made.

Increasing *m*, the number of digits requested, results in more guesses being required to identify the full PIN. This might be considered counter-intuitive, as partial PINs are intended to make it harder to reuse snooped PIN information. We have also seen that with moderate numbers of guesses (10–100 s) it is possible to recover a reasonable fraction of PINs when using the more efficient strategies. At one guess per day, a 25% success rate is possible for ${}^{4}C_{2}$ in under two months and for ${}^{6}C_{3}$ in under a year. These results, and the graphs in Section 5, may be of use to security designers who wish to understand the strength of partial PIN schemes, either individually or as part of a multi-factor scheme.

In this paper, we have mainly focused on strategies for an attacker for guessing a PIN that was assigned uniformly at random. However, PINs might be non-uniformly assigned. For example, the method of assignment of 4-digit (non-partial) PINs to ATM cards analysed by Kuhn is non-uniform and allows an attacker to identify particular PINs that have higher probability giving an approximately 0.7% chance of guessing the PIN in three guesses [10]. In this case, the non-uniformity arises from the mapping of the output from the DES cipher to decimal digits. Aspinall and Just also exploit non-uniformity in the context of partial passwords [1], however they are more focused on the situation where the non-uniformity arises because of factors such as user choice, where it is known that password choices are non-uniform [2]. In this situation, non-uniformity can provide a huge advantage. Using synthetic data based on the RockYou leak, Aspinall and Just are able to achieve over 10% coverage in a single guess! Hence the importance of advice to implement blocklists of common PINs where user-selected PINs are permitted [8].

In the introduction, we noted that our attacks correspond to an adaptive projection dictionary attack proposed in Aspinall and Just’s future work. Here *dictionary* corresponds to our PIN list, *projection* means that we use the PIN list by summarising the information at the requested digit positions and *adaptive* means that we prune the PIN list after each guess. Our results show that both Max and Educated Guess are effective, even in the case where PINs are uniformly assigned, if a moderate number of guesses are possible. We expect the advantage from these strategies can be combined with the advantages of non-uniformity. Indeed, our Max and Educated Guess strategies can actually be easily extended to the non-uniform situation by weighting each PIN with any prior information, requiring small modifications to the procedures in Algorithm 1.

We note that other attacks are possible, for example, an attacker might evesdrop on the communication and try to determine the full PIN by observing multiple successful logins. In this case, the analysis is the same as when a dictionary of passwords is available, and the distribution of successes has been calculated (see the *pure recording attack* [1]). As observed previously, incomplete information gained by evesdropping can easily be used as input to our guessing strategies via the initial set *S*.

While we have shown that our Max strategy is, in some senses, stepwise optimal, it is not clear if more effective overall strategies may exist. In addition, there may also be defensive strategies, for example adapting the digit positions requested when it is believed that an attack is ongoing. The simplest version of such a strategy might involve requesting the same digit positions until a successful login occurs. In these cases the design choices around our Max and Educated Guess strategy still hold, though the details of the performance analysis will be changed. We leave the performance and design of such defensive strategies as future work.

## 7. Conclusions

We have looked at strategies for guessing a PIN in a system where *m* digits from *n* are requested at login. We have identified two efficient strategies that make use of a PIN list. We have evaluated these strategies in the case where the PIN has 3–6 digits, providing curves that show the success rate after a number of guesses. The number of guesses increases with both *n* and *m*, though more slowly for *n*. Our results indicate it is often possible to have a moderate chance of recovering the full PIN with tens to hundreds of guesses.

## Figures and Tables

**Figure A1:**
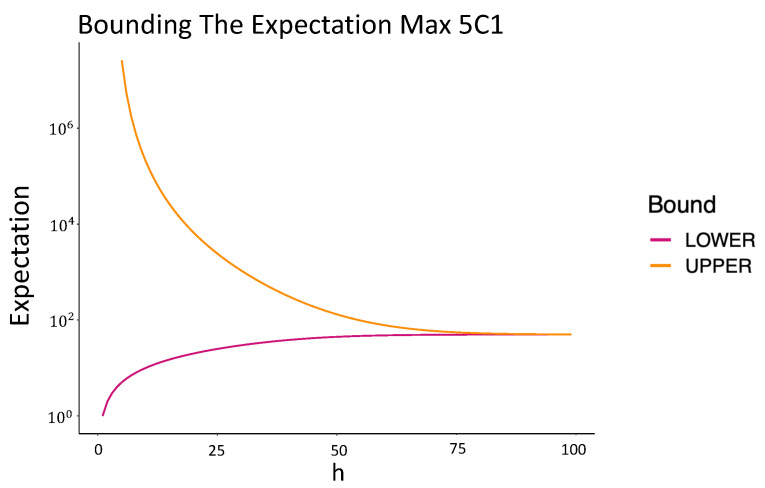
Bounding the expectation number of guesses for Max ${}^{5}C_{1}$.

## Appendix A

### Appendix A.1. Max Strategy

Here we show how to derive the distribution of number of guesses using the Max strategy for ${}^{n}C_{n}$ and ${}^{n}C_{1}$ problems.

#### Appendix A.1.1. Max Strategy for ${}^{n}C_{n}$ Problems

The expression for this problem is relatively easy to derive, but will be useful for studying the ${}^{n}C_{1}$ problem. Suppose we are following the Max strategy, and we are guessing a ${}^{n}C_{n}$ problem. Each guess involves all *n* digits, and so tells us if one PIN is on the list. As all PINs are equally probable, we essentially eliminate one possibility among $10^{n}$ each time. Thus, the probability that we are correct within the first *k* guesses is simply

(A1) $$P\left[\mathrm{correct}\;\mathrm{in}\;k\;\mathrm{guesses}\right]=\min\left(\frac{k}{10^{n}},1\right).$$

Or, if *G* is the number of guesses required,

$$P\left[G>k\right]=1-\min\left(\frac{k}{10^{n}},1\right)=\max\left(1-\frac{k}{10^{n}},0\right).$$

and so, by the Tail Sum Formula, the expected number of guesses is

(A2) $$E\left[G\right]=\sum_{k=0}^{\infty }P\left[G>k\right]=\sum_{k=0}^{\infty }\max\left(1-\frac{k}{10^{n}},0\right)=\sum_{k=0}^{10^{n}}1-\frac{k}{10^{n}}=\frac{10^{n}+1}{2}.$$

We note that for an ${}^{n}C_{n}$ problem, recovering the full PIN is essentially the same as achieving a successful guess.

#### Appendix A.1.2. Max Strategy for ${}^{n}C_{1}$ Problems

Now, suppose that we are using the Max strategy on a ${}^{n}C_{1}$ problem, so each guess tells us about just one digit, and we can maintain independent lists for each digit. First consider the case where we have made *k* guesses in total, with $k_{i}$ guesses made for each digit *i*.

$$P\left[G>k|k_{i}\;\mathrm{guesses}\;\mathrm{of}\;\mathrm{digit}\;i\right]=1-P\left[\mathrm{all}\;\mathrm{digits}\;\mathrm{correct}|k_{i}\;\mathrm{guesses}\;\mathrm{of}\;\mathrm{digit}\;i\right],$$

but, as the guesses for digit *i* are independent

$$P\left[G>k|k_{i}\;\mathrm{guesses}\;\mathrm{of}\;\mathrm{digit}\;i\right]=1-\prod_{i=1}^{n}P\left[\mathrm{digits}\;i\;\mathrm{correct}|k_{i}\;\mathrm{guesses}\;\mathrm{of}\;\mathrm{digit}\;i\right].$$

Now, we can treat each digit as a ${}^{1}C_{1}$ problem and, using Equation (A1) with n=1, we get

$$P\left[G>k|k_{i}\;\mathrm{guesses}\;\mathrm{of}\;\mathrm{digit}\;i\right]=1-\prod_{i=1}^{n}\min\left(\frac{k_{i}}{10},1\right).$$

Now, given that we have made *k* guesses in total, if the digits guessed were randomly chosen, we can also find the probability that we have made $k_{i}$ guesses for digit *i*. Naturally $k_{1}+k_{2}+\ldots +k_{n}=k$. Using the multinomial distribution, the probability of this is

kk1k2…kn1nk1…1nkn=kk1k2…kn1nk.

Then, by the law of total probability, we can find P[G>k] as

∑k1+…+kn=kP[G>k|ki guesses of digit i]kk1k2…kn1nk.

So, by the Law of Total Probability, we find,

P[G>k]=∑k1+…+kn=k1−∏i=1nminki10,1kk1k2…kn1nk.

or, slightly simplifying using the multinomial theorem,

(A3) P[G>k]=1−1nk∑k1+…+kn=kkk1k2…kn∏i=1nminki10,1.

We may then find E[G] by summing this over *k*.

(A4) E[G]=∑k=0∞P[G>k]=∑k=0∞1−1nk∑k1+…+kn=kkk1k2…kn∏i=1nminki10,1.

We briefly note that the inner sum is a effectively a sum over compositions of *n*, as if any $k_{i}=0$, then the whole term is zero. Also note that the terms are symmetric in the $k_{i}$, and so this sum over compositions could be written as a sum over partitions, by accounting for how many compositions correspond to each partition.

We note that a similar calculation can be used to find the distribution of times to first successful guess. First, observe that the chance of the first success happening in *k* guesses is the complement of there being no successes in *k* guesses. Then note that that is independent for each digit being guessed, and so a product and the conditioning on $k_{i}$ guess for digit *i* can be used.

### Appendix A.2. Random Strategy

Here we derive the distribution of number of guesses using the Random strategy for ${}^{n}C_{n}$ and ${}^{n}C_{1}$ problems, using a similar method to that used for the Max strategy. While we are focused on the number of guesses to recover the full PIN, we note that for the random strategy for an ${}^{n}C_{m}$ problem, the distribution of the number of guesses before the first success is particularly simple, as each guess is effectively guessing an *m* digit number using no state, so the distribution will be geometric.

#### Appendix A.2.1. Random Strategy for ${}^{n}C_{n}$ Problems

Suppose we are looking at an ${}^{n}C_{n}$ problem, and as we use the Random strategy each guess is uniformly random, ignoring our previous choices. We will stop guessing if we either choose the correct pin or we choose every incorrect PIN. After guess *k*, the chance that we have never chosen the correct PIN is

$${\left(1-\frac{1}{10^{n}}\right)}^{k}.$$

If $k<10^{n}-1$, then the chance of having selected all incorrect PINs is zero. Thus, if $k<10^{n}-1$, and again *G* is the random variable for the number of guesses required to guess correctly, we have

(A5) $$P\left[G>k\right]={\left(1-\frac{1}{10^{n}}\right)}^{k}$$

If $k\geq 10^{n}-1$, then there is a possibility that we have guessed all the incorrect numbers, and we need to make a correction:

(A6) P[G>k]=1−110nk1−k10n−110n−1!10n−1k,

where the numerator of the second term uses Stirling numbers of the second kind to count partitions of *k* into non-empty sets corresponding to each of the $10^{n}-1$ incorrect digits, and the denominator is the number of ways of assigning *k* choices to *any* subset of the $10^{n}-1$ incorrect possibilities. Note, in both cases we have

$$P\left[G>k\right]\leq {\left(1-\frac{1}{10^{n}}\right)}^{k}.$$

Now, we can calculate the expected number of guesses and also upper bound it.

(A7) $$E\left[G\right]=\sum_{k=1}^{\infty }P\left[G\geq k\right]=\sum_{k=0}^{\infty }P\left[G>k\right]\leq \sum_{k=0}^{\infty }{\left(1-\frac{1}{10^{n}}\right)}^{k}=10^{n}.$$

#### Appendix A.2.2. Random Strategy for ${}^{n}C_{1}$ Problems

Now, suppose we are looking at ${}^{n}C_{1}$ with the Random strategy. On guess *k* we will stop guessing if we have correctly guessed each digit, or we have eliminated all digits except the correct one. Again, let’s condition on having made $k_{i}$ guesses for digit *i* and naturally $k_{1}+k_{2}+\ldots +k_{n}=k$. As we saw for the Max strategy case, given there have been $k_{i}$ guessed for digit *i*, the probability what we are still guessing is the probability that not all digits are known,

$$1-P\left[\mathrm{all}\;\mathrm{digits}\;\mathrm{known}\right]=1-\prod_{i=1}^{n}P\left[\mathrm{digits}\;i\;\mathrm{known}\right]=1-\prod_{i=1}^{n}1-P\left[G_{i}>k_{i}\right],$$

where $G_{i}$ is the number of guesses taken to guess digit *i*. By treating each digit as a ${}^{1}C_{1}$ problem, we can use Equation (A6) with n=1 we can get $P[G_{i}>k_{i}]$. We can then sum this using the law of total probability, to get

P[G>k]=∑k1+…+kn=k1−∏i=1n1−P[Gi>ki]kk1k2…kn1nk.

Or, tidying using the multinomial theorem,

(A8) P[G>k]=1−1nk∑k1+…+kn=kkk1k2…kn∏i=1n1−P[Gi>ki].

Again, we can use the Tail Sum Formula to get

(A9) E[G]=∑k=0∞1−1nk∑k1+…+kn=kkk1k2…kn∏i=1n1−P[Gi>ki].

Using the same observations as above, the inner sum could be written as a sum over partitions rather than compositions.

#### Appendix A.2.3. Bounding the Expectation

In the cases of the Max and Random ${}^{n}C_{n}$ and ${}^{n}C_{1}$ problems we have found P[G>k], and then used the Tail Sum Formula to find E[G] via an infinite sum. For our guessing strategies, there is a useful way to evaluate only a finite number of terms while still bounding E[G] above and below.

First, consider a modified guessing process, where we choose a constant *h* and make up to *h* guesses using the original strategy. If we are successful on any guess, we stop guessing. However, if we reach guess *h* we stop regardless of if we are successful on guess *h*. Let $H_{h}$ be the random variable representing the number of steps this process takes to stop. Clearly, $H_{h}\leq G$ for any set of guesses, so $E\left[H_{h}\right]\leq E\left[G\right]$. We also note that if k<h then $P\left[H_{h}>k\right]=P\left[G>k\right]$ and $P[H_{h}>k]=0$ for k≥h. This means that we can find

(A10) $$E\left[H_{h}\right]=\sum_{k=0}^{h-1}P\left[G>k\right],$$

and use this as a lower bound for E[G] for the original strategy. We also note that the probability that this process failed to make the correct guess is just P[G>h].

Next, consider a second modified guessing process which takes up to *h* guesses using the strategy, but then forgets everything that it has learned if it has not found the correct PIN (say, by restoring all possibilities to the PIN list). This forgetting happens after every block of *h* guesses. Let $F_{h}$ be the number of steps required before modified this process finds the correct PIN. Compared to any reasonable strategy, this modified process should be less efficient than the unmodified strategy, so $E\left[G\right]\leq E\left[F_{h}\right]$. Under the assumption that success in each block of *h* guesses is independent, we can use the law of total expectation to write

$$\begin{array}{cc} \; & E[F_{h}]\\ \; & =\sum_{k=1}^{\infty }E\left[\#\mathrm{guesses}|\mathrm{success}\;\mathrm{in}\;k^{th}\;\mathrm{block}\right]\times P\left[\mathrm{successful}\;\mathrm{in}\;k^{th}\;\mathrm{block}\right],\\ \; & =\sum_{k=1}^{\infty }\left((k-1)h+E[\#\mathrm{guesses}|\mathrm{successful}\;\mathrm{in}\leq h\;\mathrm{guesses}]\right)\times P{\left[G>h\right]}^{k-1}\left(1-P\left[G>h\right]\right),\\ \; & =h\frac{P[G>h]}{1-P[G>h]}+E\left[\#\mathrm{guesses}|\mathrm{successful}\;\mathrm{in}\leq h\;\mathrm{guesses}\right]. \end{array}$$

However, observe that

$$E\left[\#\mathrm{guesses}|\mathrm{successful}\;\mathrm{in}\leq h\;\mathrm{guesses}\right]=\frac{\sum_{i=0}^{h}P\left[G=i\right]i}{1-P[G>h]},$$

and the sum in the numerator is bounded above by $E[H_{h}]$. So using $E\left[H_{h}\right]\leq E\left[G\right]\leq E\left[F_{h}\right]$ and Equation (A10) we get convenient bounds:

(A11) $$\sum_{k=0}^{h-1}P\left[G>k\right]\leq E\left[G\right]\leq \frac{1}{1-P[G>h]}\left(hP\left[G>h\right]+\sum_{k=0}^{h-1}P\left[G>k\right]\right).$$

As the bounds are all in terms of quantities that we will calculate when evaluating the Tail Sum for E[G], they provide useful guidance when truncating the infinite sum when evaluating the sum in practice. This can be particularly useful for the sums in Equations (A4) and (A9), which involve summing over increasingly large compositions/partitions. An example of these bounds is shown in Figure A1 for calculating the mean of the Max ${}^{5}C_{1}$ problem. We see that the bound is quite tight after approximately 70 terms.

#### Appendix A.2.4. Round Robin Simulation Results

For completeness, we include the results of simulations for the Round Robin strategy in Figure A2. These results are the analogue of those shown in Figure 4. As noted, because of the simple construction of the Round Robin strategy, it will not usually successfully guess the ${}^{n}C_{n}$ problems, so no corresponding result is shown.

## References

[1] Aspinall D., Just M. (2013). “Give me letters 2, 3 and 6!”: Partial password implementations and attacks. Proceedings of the International Conference on Financial Cryptography and Data Securitym.

[2] Malone D., Maher K. Investigating the distribution of password choices. Proceedings of the 21st International Conference on World Wide Web.

[3] Bonneau J. (2012). Guessing Hu man-cho Sen Secrets. Ph.D. Thesis.

[4] Markert P., Bailey D.V., Golla M., Dürmuth M., Aviv A.J. This PIN Can Be Easily Guessed: Analyzing the Security of Smartphone Unlock PINs. Proceedings of the IEEE Symposium on Security and Privacy.

[5] Wolters P., Jacobs B. (2019). The security of access to accounts under the PSD2. Comput. Law Secur. Rev..

[6] Banking & Payments Federation Ireland. PSD2 Is Coming. Are You Ready?. https://www.youtube.com/watch?v=XrALDRsaI-M.

[7] Mourouzis T., Wojcik M., Komninos N. (2016). On the security evaluation of partial password implementations. arXiv.

[8] Bonneau J., Preibusch S., Anderson R. (2012). A birthday present every eleven wallets? The security of customer-chosen banking PINs. Proceedings of the International Conference on Financial Cryptography and Data Security.

[9] Bentley J., Mallows C. (2005). How much assurance does a PIN provide?. Proceedings of the International Workshop on Human Interactive Proofs.

[10] Kuhn M. (1997). Probability theory for pickpockets—ec-PIN guessing. Proceedings of the Workshop on Cryptography and Network Security.

[11] Knuth D.E. (1976). The computer as Master Mind. J. Recreat. Math..

[12] Focardi R., Luccio F.L. (2012). Guessing bank PINs by winning a mastermind game. Theory Comput. Syst..

[13] Chiasson S., Van Oorschot P.C. (2015). Quantifying the security advantage of password expiration policies. Des. Codes Cryptogr..

[14] Christiansen M.M., Duffy K.R. (2012). Guesswork, large deviations, and Shannon entropy. IEEE Trans. Inf. Theory.

[15] Duffy K.R. (2021). Guesswork. Lond. Math. Soc. Newsl..

[16] Li J. (2019). Large deviations for conditional guesswork. Stat. Probab. Lett..

